# Transcriptional regulation of flavonoid biosynthesis in nectarine (*Prunus persica*) by a set of R2R3 MYB transcription factors

**DOI:** 10.1186/1471-2229-13-68

**Published:** 2013-04-25

**Authors:** Daniela Ravaglia, Richard V Espley, Rebecca A Henry-Kirk, Carlo Andreotti, Vanina Ziosi, Roger P Hellens, Guglielmo Costa, Andrew C Allan

**Affiliations:** 1 Department of Fruit Tree and Woody Plant Sciences, Alma Mater Studiorum, University of Bologna, Viale Fanin 46, 40127, Bologna, Italy; 2 The New Zealand Institute for Plant and Food Research (PFR), Private Bag 92 169, Auckland, New Zealand; 3 Faculty of Science and Technology, Free University of Bozen, Piazza Università 5, Bozen, 39100, Italy; 4 School of Biological Sciences, University of Auckland, Private Bag 92 019, Auckland, New Zealand

**Keywords:** Anthocyanin, Transcriptional regulation, MYB, Peach, Nectarine, *Prunus persica*, Light

## Abstract

**Background:**

Flavonoids such as anthocyanins, flavonols and proanthocyanidins, play a central role in fruit colour, flavour and health attributes. In peach and nectarine (*Prunus persica*) these compounds vary during fruit growth and ripening. Flavonoids are produced by a well studied pathway which is transcriptionally regulated by members of the MYB and bHLH transcription factor families. We have isolated nectarine flavonoid regulating genes and examined their expression patterns, which suggests a critical role in the regulation of flavonoid biosynthesis.

**Results:**

In nectarine, expression of the genes encoding enzymes of the flavonoid pathway correlated with the concentration of proanthocyanidins, which strongly increases at mid-development. In contrast, the only gene which showed a similar pattern to anthocyanin concentration was *UDP-glucose-flavonoid-3-O-glucosyltransferase* (*UFGT)*, which was high at the beginning and end of fruit growth, remaining low during the other developmental stages. Expression of *flavonol synthase* (*FLS1)* correlated with flavonol levels, both temporally and in a tissue specific manner. The pattern of *UFGT* gene expression may be explained by the involvement of different transcription factors, which up-regulate flavonoid biosynthesis (*MYB10*, *MYB123*, and *bHLH3*), or repress (*MYB111* and *MYB16*) the transcription of the biosynthetic genes. The expression of a potential proanthocyanidin-regulating transcription factor, *MYBPA1*, corresponded with proanthocyanidin levels. Functional assays of these transcription factors were used to test the specificity for flavonoid regulation.

**Conclusions:**

MYB10 positively regulates the promoters of *UFGT* and *dihydroflavonol 4-reductase* (*DFR*) but not *leucoanthocyanidin reductase* (*LAR*). In contrast, MYBPA1 trans-activates the promoters of *DFR* and *LAR*, but not *UFGT*. This suggests exclusive roles of anthocyanin regulation by MYB10 and proanthocyanidin regulation by MYBPA1. Further, these transcription factors appeared to be responsive to both developmental and environmental stimuli.

## Background

Anthocyanins, flavonols and flavan-3-ols belong to the group of the ubiquitous secondary metabolites known as flavonoids. They represent the main classes of phenolic compounds in fruit [1-3] and play a central role as determinants of fruit quality. The accumulation of anthocyanin pigments in fruit provides essential cultivar differentiation for consumers and represents an important factor for marketability, while the flavan-3-ols (precursors of the proanthocyanidins) can be a major influence on fruit flavour. Proanthocyanidins impart astringency to fresh fruits, fruit juices and wine, oxidise to form brown pigments in seeds and other tissues and may act as feeding deterrents in reproductive tissues and developing fruits [4,5]. Anthocyanins, flavonols and flavan-3-ols influence the health attributes of our fruit thanks to their natural antioxidant capacity [6]. Several studies correlated the intake of these compounds with the lower incidences of certain chronic diseases such as cancers, cardiovascular, and neurodegenerative disease [7-11].

Peach is an important global *Rosaceous* fruit with world production of over 18 million tonnes in 2009, concentrated in centres such as China, Italy, Spain and USA (http://www.fao.org). Peach and nectarine (both *Prunus persica)* are characterized by a wide range of different cultivars, with their health attributes and colour of both skin and flesh being important factors for consumer choice. Red colour in peach fruit has been the object of most breeding programs. In particular, high levels of red colouration have been sought in cultivars for the fresh market. In contrast, reduced pigment content in any part of the fruit has been the aim of most breeding programs for the canning industry because of the possibility of staining in the processed product. The primary pigment responsible for red colouration in peaches and nectarines is cyanidin and, in particular, cyanidin 3-glucoside, one of the most common anthocyanin pigments in fruit [12,13]. The main flavan-3-ol identified in peach is catechin, while the flavonols are dominated by three glycosylated quercetins (quercetin 3-galactoside, quercetin 3-glucoside and quercetin 3-rutinoside) [12,13].

**Figure 1:**
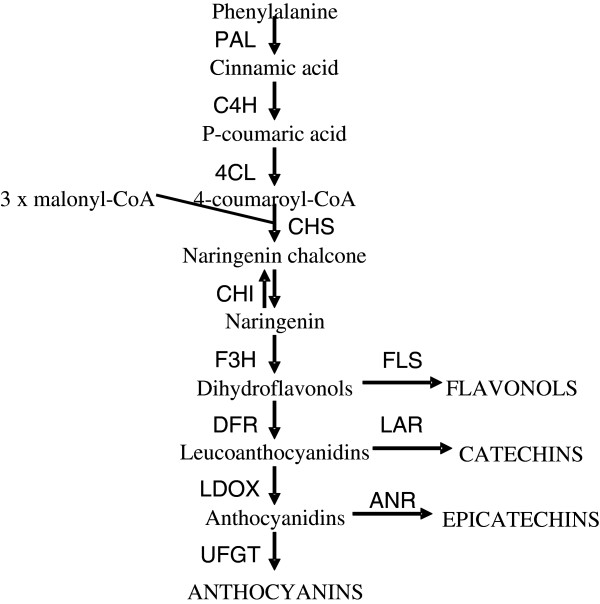
**Scheme of the flavonoid biosynthetic pathway in plants.** Genes encoding enzymes for each step are indicated as follows: *PAL, phenylalanine ammonia-lyase; C4H, cinnamate 4-hydroxylase; 4CL, 4-coumarate-CoA ligase; CHS, chalcone synthase; CHI, chalcone isomerase; F3H, flavanone 3-hydroxylase; DFR, dihydroflavonol 4-reductase; LDOX, leucoanthocyanidin dioxygenase; FLS, flavonol synthase; LAR, leucoanthocyanidin reductase; ANR, anthocyanidin reductase; UFGT, UDP-glucose:flavonoid-3-O-glycosyltransferase.*

Anthocyanins, flavonols and flavan-3-ols are synthesized via the flavonoid pathway, whose genetics and biochemistry have been well characterized in several plant species [14,15]. The pathway consists of several steps, which are common to the synthesis of different flavonoids, and branch steps, which are more specific for each type of flavonoid (Figure 1). The flavonoid pathway appears to be mainly regulated at the level of transcription of genes encoding the enzymes of the pathway [15]. Several transcription factors (TFs) have been isolated in a diverse group of plants [16-20] which control this transcription. In particular, interacting R2R3-MYB and bHLH type TFs, form a complex with WD40 proteins (termed the MBW complex), to activate the anthocyanin and proanthocyanidin biosynthetic genes. The MBW complex usually regulates groups of flavonoid biosynthetic genes, which vary between species [17]. This regulation is via specific binding to motifs in the promoters of the pathway genes [21-23].

Several studies conducted on apple (*Malus* × *domestica*) confirmed that all the biosynthetic genes leading to the anthocyanin synthesis are co-ordinately regulated [24-26]. The R2R3-MYB and bHLH TFs responsible for the anthocyanin accumulation have been isolated and characterized [27-30]. In contrast, the grape R2R3-MYB TF VvMYBA, which regulates anthocyanin biosynthesis, appears to activate only the *UDP-glucose:flavonoid-3-O-glycosyltranferase* (*UFGT*) gene. This plays a key role in colour development in grape skin, distinguishing white and red grapes [31,32]. A peach TF, PpMYB10, has been shown to activate both the tobacco anthocyanin pathway, and the *Arabidopsis dihydroflavonol 4-reductase* (*DFR)* promoter, when assayed transiently [33].

Flavonols are synthesized from the dihydroflavonols by flavonol synthase (FLS) enzymes (Figure 1). In peach the major flavonols are quercetin derivatives [13]. In *Arabidopsis*, the regulation of flavonol biosynthesis has been investigated. Three closely related MYBs AtMYB11, AtMYB12 and AtMYB111 (also known as PFG1–PFG3, for Production of Flavonol Glucosides) regulate AtFLS1 and other steps [34,35]. In maize, the orthologue of FLS is regulated by UV exposure via activation of maize’s MYB/bHLH orthologous transcription factors [36]. In grape VvMYBF1 regulates grape *FLS* in a light dependent manner [37].

The genes encoding enzymes specific for the proanthocyanidins (*ANR: anthocyanidin reductase; LAR: leucoanthocyanidin reductase*) have been isolated from, amongst others, apple and grape [38-40]. Takos et al., (2006) [39] showed that the proanthocyanidin biosynthetic genes in apple appear to be differentially regulated in comparison to other flavonoid biosynthetic genes. Studies in grape report the characterization of at least four MYB TFs (VvMYB5a, VvMYB5b, VvMYBPA1 and VvMYBPA2) that regulate steps in the flavonoid pathway, which could affect the accumulation of the proanthocyanidins in leaves, flowers and early in berry development, before the véraison stage. These MYBs induce the expression of both the proanthocyanidin branch genes and several of the general flavonoid pathway genes, but not *UFGT*[41].

Adding to the complexity of the transcriptional control of flavonoid biosynthesis is a group of MYB TFs which act as repressors of the pathway [42,43]. In particular, the regulatory activity of an R3-MYB TF called AtMYBL2 (At1g71030) has been characterized in *Arabidopsis,* where it plays a crucial role in the repression of anthocyanin biosynthesis. A model has been proposed whereby AtMYBL2 interferes with the activity of the MBW complex [44,45]. In apple [46] and strawberry [42] MYB repressors have been examined and shown to increase with fruit maturity, even though anthocyanin levels are increasing, suggesting competition with MYB activators.

Most of the genes encoding enzymes leading to the anthocyanin synthesis in peach fruit have been studied [47,48]. Crosses of red and non-red peaches suggest that a major gene controls peach colour [49,50]. However, there is no data about either the flavonol (*FLS*) or flavan-3-ol specific genes (*ANR* and *LAR*) or the regulation of the flavonoid metabolism in this plant. In this study we report the transcript levels of the main biosynthetic genes leading to the synthesis of anthocyanins, flavonols, and flavan-3-ols in ‘Stark Red Gold’ nectarines during fruit growth, ripening, and environmental manipulation. We also identified key TFs, which appear to play a crucial role in the regulation of the flavonoid pathway in peach/nectarine acting to activate (*MYB10, MYB123*, *bHLH3, WD40* and *MYBPA1*) or potentially repress (*MYB16* and *MYB111*). Members of these transcriptional complexes activated the peach *UFGT* and *LAR1* promoters, and were responsive to both developmental and environmental stimuli.

## Results

### Anthocyanin and flavan-3-ol accumulation during fruit development and ripening

**Figure 2:**
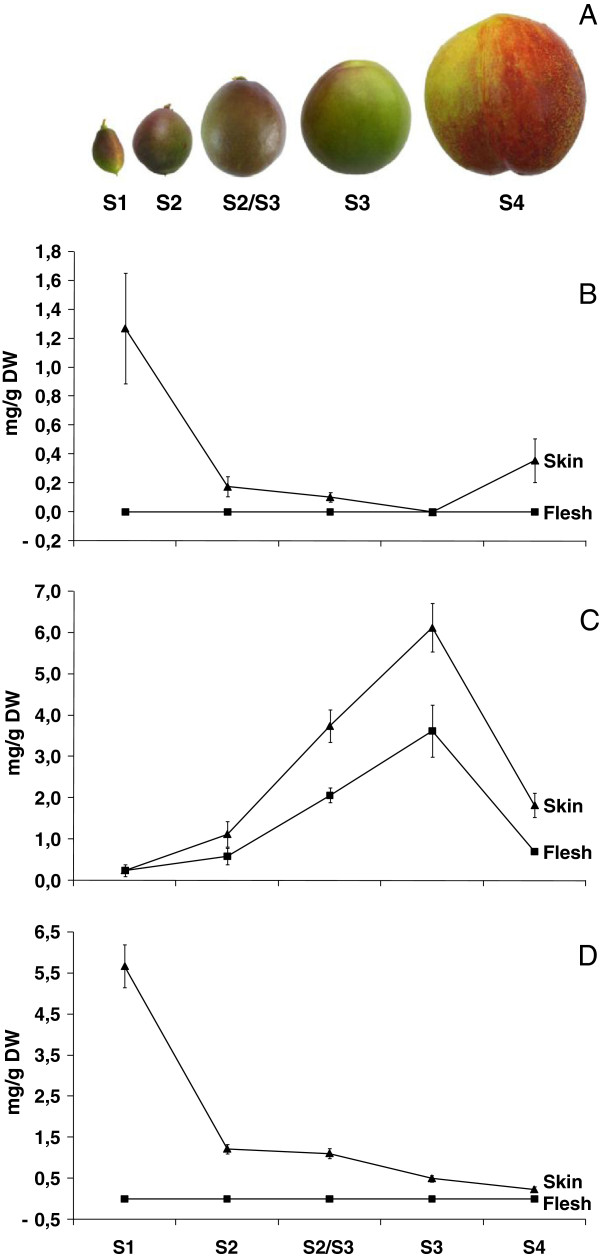
**Fruit development series of ‘Stark Red Gold’ nectarines (A) and their anthocyanin (B) and proanthocyanidin (C) and (D) flavonol concentration (mg g**^**-1 **^**dry weight).** The developmental stages are indicated as follows: S1, 50 DAFB; S2, 66 DAFB; S2-3, 74 DAFB; S3, 90 DAFB; S4, 135 DAFB. Error bars are standard deviation of the mean for five replicates.

The development series of ‘Stark Red Gold’ nectarine, and the anthocyanin and flavan-3-ol accumulation in fruit tissues, are shown in Figure 2. During fruit growth there were two peaks of anthocyanin accumulation in peel: one early in development (50 DAFB) and the other at the end of fruit growth (135 DAFB; Figure 2B). Very low concentrations of anthocyanin were observed for fruit in the middle stages of development. The anthocyanins detected were either cyanidin 3-glucoside or cyanidin 3-rutinoside as seen in previous studies [13], with cyanidin 3-rutinoside present only at the beginning of fruit development (50 DAFB) at low concentration. In contrast, the concentration of proanthocyanidins or flavan-3-ols, in both peel and flesh, was low at the beginning of fruit development (50 DAFB), strongly increased at mid-development, and finally decreased again during ripening (135 DAFB; Figure 2C). Flavonols (mainly quercetin-3-galactoside with some quercetin-3-glucoside and quercetin-3-rutinoside) showed a similar pattern to anthocyanin, being skin located, but showed no increase at maturity (Figure 2D).

No anthocyanin was detected by HPLC analysis in the flesh at any developmental stage. However, a few red stripes were observed near the stone during the sampling of the ripe fruit (S4). This suggests that this cultivar can accumulate anthocyanins in flesh, but in the sampled tissue concentrations were too low for detection by HPLC analysis.

### RNA expression profile of the flavonoid biosynthetic enzymes in nectarine fruit

**Figure 3:**
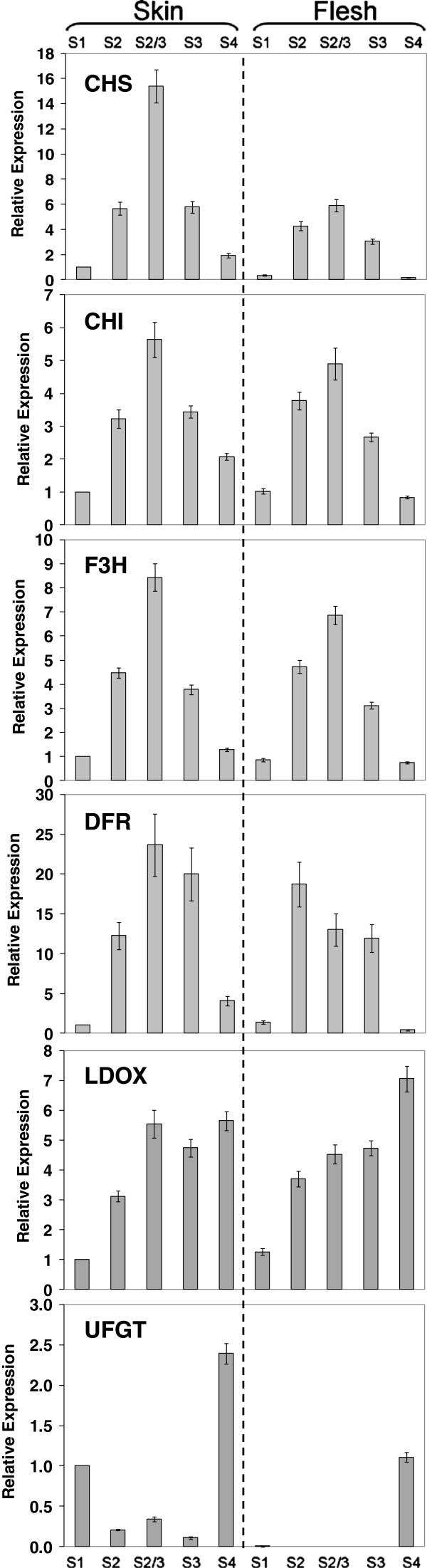
**Relative expression profiling of biosynthetic genes for main pathway flavonoid and anthocyanin synthesis in skin and flesh of ‘Stark Red Gold’ nectarines.** The developmental stages are indicated as follows: S1, 50 DAFB; S2, 66 DAFB; S2-3, 74 DAFB; S3, 90 DAFB; S4, 135 DAFB. *PpActin* used as reference gene. Error bars are standard error of the mean for three replicate reactions.

**Figure 4:**
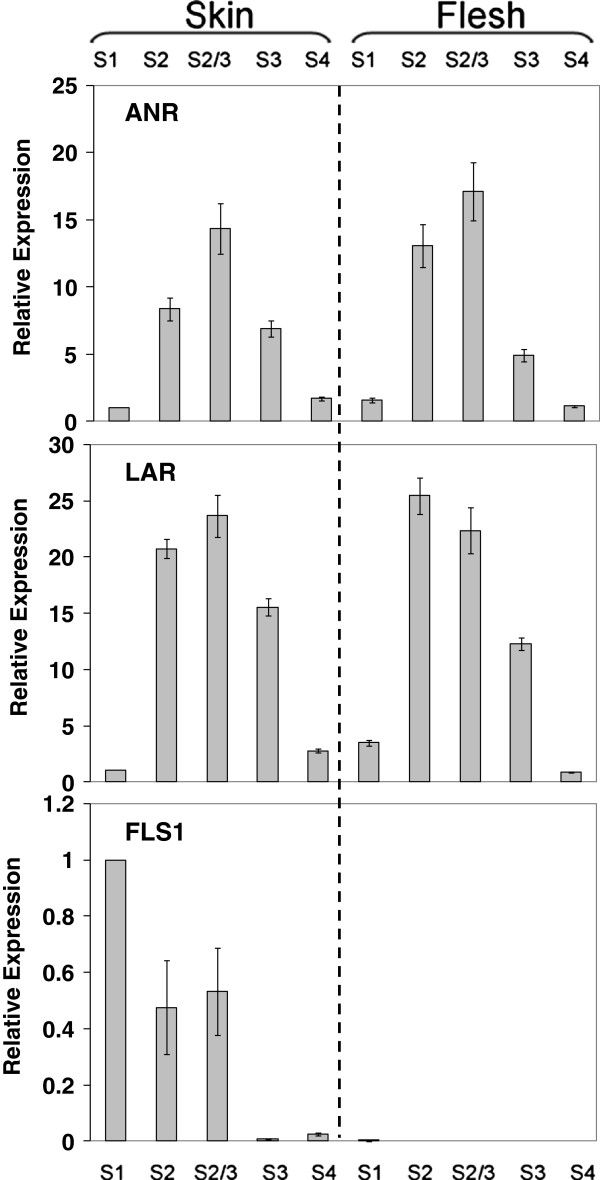
**Relative expression profiling of biosynthetic genes for branch pathway flavonol and proanthocyanidin synthesis in skin and flesh of ‘Stark Red Gold’ nectarines.** The developmental stages are indicated as follows: S1, 50 DAFB; S2, 66 DAFB; S2-3, 74 DAFB; S3, 90 DAFB; S4, 135 DAFB. *PpActin* used as reference gene. Error bars are the standard error of the mean for three replicate reactions.

The sequences of nine putative flavonoid biosynthetic genes were identified in the publicly available peach nucleotide and EST databases. For each biosynthetic gene, we selected the peach sequence expressed in fruit which showed the highest homology in BLAST searches to the *Malus* × *domestica* corresponding protein. The levels of transcription of these nine selected flavonoid biosynthetic genes were determined, in both peel and flesh of ‘Stark Red Gold’ nectarines, during fruit development and ripening using quantitative PCR (qPCR, Figures 3 and 4).

The early biosynthetic genes common to both anthocyanin and flavan-3-ol biosynthesis within the phenylpropanoid pathway (*CHS*, *CHI*, *F3H*, *DFR*) and the late biosynthetic steps specific for proanthocyanidins and anthocyanin (*LDOX* and *UFGT*) showed contrasting patterns (Figure 3). The transcript levels of most of these genes were very low at the beginning of fruit development (50 DAFB), strongly increased up to 74 DAFB and then decreased again through to fruit maturation (135 DAFB) in both peel and flesh. A slightly different expression profile was recorded for the *LDOX* gene, which encodes the enzyme that is the last common step shared by anthocyanin and flavan-3-ol biosynthesis. Its transcript level in both peel and flesh was low at the beginning of fruit development, increased from 66 DAFB up to 74 DAFB, and then remained high throughout fruit maturation (Figure 3).

In contrast, the expression profile of the *UFGT* gene was significantly different to all the other biosynthetic genes examined (Figure 3). Its transcript in peel was most abundant at the beginning (50 DAFB) and end (135 DAFB) of fruit growth, remaining low throughout the middle of development (from 66 to 90 DAFB). *UFGT* transcript was detected also in flesh, but only in the last ripe fruit time point (135 DAFB). This pattern of expression correlated with the pattern of anthocyanin accumulation in fruit, but was not statistically significant (Additional file 1: Table S1).

Transcript levels of *FLS* were well correlated with the accumulation of flavonols (Figure 4). *FLS* showed no flesh expression suggesting tight skin-specific regulation of the gene. *LAR* and *ANR* showed very similar patterns of expression to each other and were expressed in both skin and flesh. This followed the profile of proanthocyanidin levels (Figure 2) which is present in both skin and flesh.

### Quantitative PCR expression analysis of putative transcription factor genes in nectarine fruit

**Figure 5:**
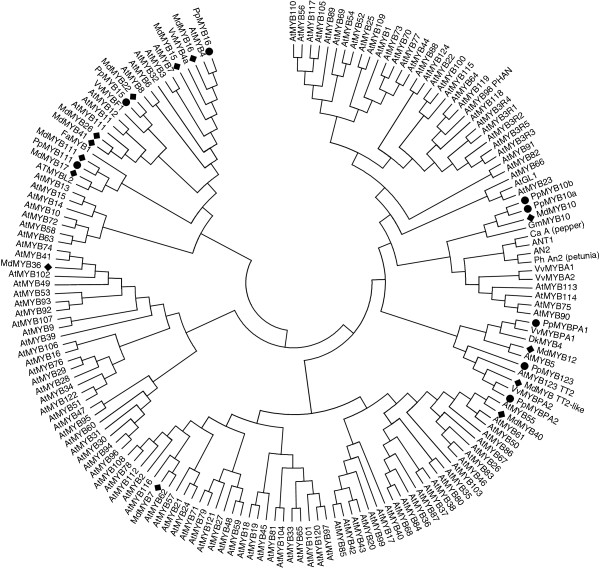
**Phylogenetic relationships between *Arabidopsis *MYB transcription factors and polyphenolic-related MYBs of other species.** Sequences were aligned using Clustal W (opening = 15, extension = 0.3) in Vector NTI 9.0. Phylogenetic and molecular evolutionary analysis was conducted using MEGA version 3.1 [51] using minimum evolution phylogeny test and 1000 bootstrap replicates.

Candidate genes encoding TFs involved in the regulation of the flavonoid biosynthesis were selected within *Prunus* sequences in the Genome Database for Rosaceae (http://www.rosaceae.org) or by homology with *Malus* × *domestica* and *Arabidopsis* TFs. In particular, the sequences showing the highest homology to members of the anthocyanin regulating transcriptional complex (MBW) and polyphenolic-related MYBs were selected for primer design. Lead candidates for expression analysis were peach *MYB10* and *bHLH3,* because of their high homology to the activators of anthocyanin synthesis in apple (*MdMYB10* and *MdbHLH3*). A candidate gene showing high BLAST homology to the WD40 TTG1 (At5g24520) protein was found (ACQ65867). R2R3 MYBs with homology to *VvMYBF1*, *VvMYB5a*, *AtMYB123* (*TT2*), *VvMYBPA2* and *VvMYBPA1* were selected by best blast match. Potential repressor MYBs, peach *MYB111* and *MYB16* showed homology to apple repressors [46]. A phylogeny of full length predicted proteins was generated (Figure 5).

**Figure 6:**
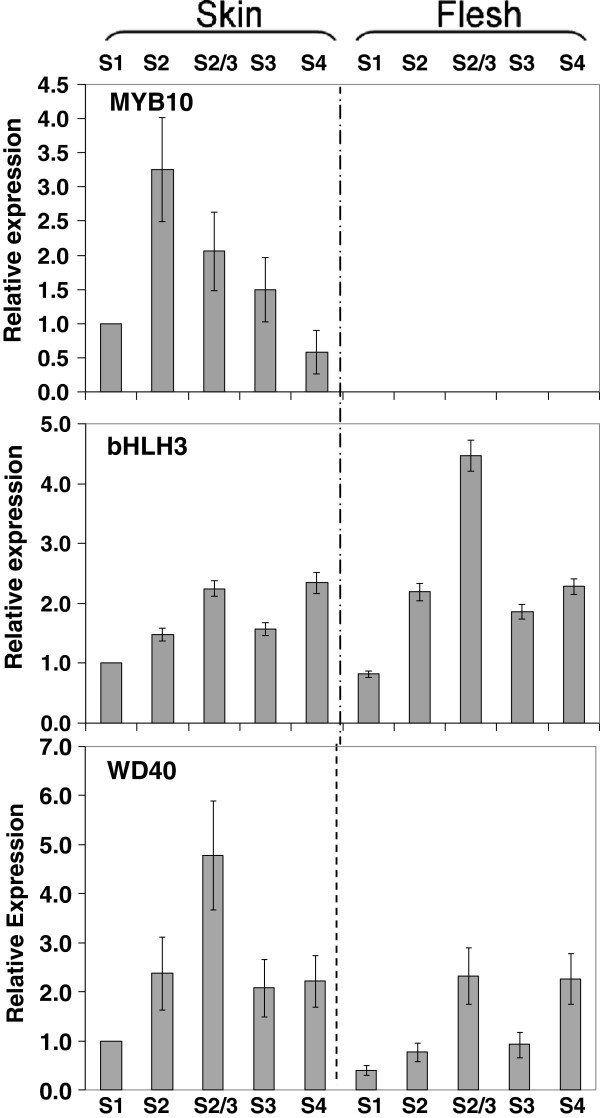
**Relative expression analyses of the transcripts of *MYB10*, *bHLH3 *and *WD40 *in peel and flesh of ‘Stark Red Gold’ nectarines during development and ripening.** The developmental stages are indicated as follows: S1, 50 DAFB; S2, 66 DAFB; S2-3, 74 DAFB; S3, 90 DAFB; S4, 135 DAFB. Error bars are the standard error of the mean of three replicate reactions.

The expression profiles of candidate TF genes, *MYB10*, *bHLH3* and *WD40*, were investigated in both peel and flesh of ‘Stark Red Gold’ nectarines at different developmental stages (Figure 6). Transcript of *MYB10* was detectable only in fruit peel, where its level was highest from 66 to 90 DAFB and lower at the beginning (50 DAFB) and end of fruit growth (135 DAFB). Two notable features of the *MYB10* profile were the absence at all stages of any flesh *MYB10* transcript and the lower levels of transcript in mature skin at a stage when *UFGT* and anthocyanin accumulation is at its maximum. Expression profiles of the *bHLH3* and *WD40* genes in both peel and flesh followed the pattern of most of the genes encoding biosynthetic enzymes, with a maximum at 90 DAFB in fruit development. Its transcript was also well expressed in ripe fruit (135 DAFB), as was observed for the *LDOX* and *UFGT* genes.

**Figure 7:**
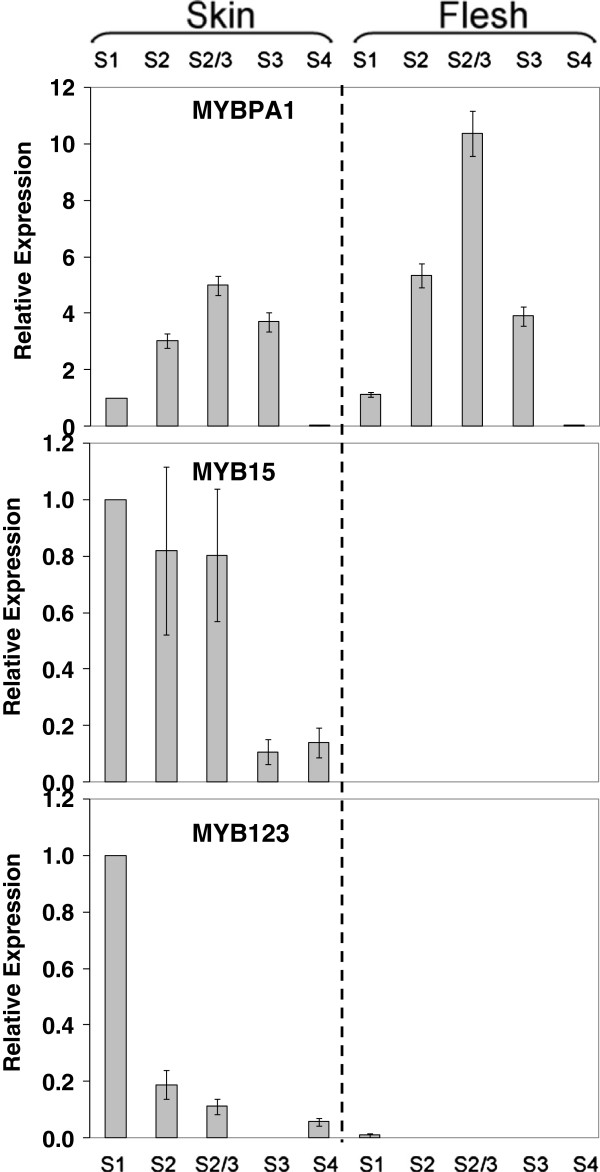
**Relative expression analyses of the transcripts of the potential MYB flavonoid activators, *MYBPA1*, *MYB15 *and *MYB123 *in peel and flesh of ‘Stark Red Gold’ nectarines during development and ripening.** The developmental stages are indicated as follows: S1, 50 DAFB; S2, 66 DAFB; S2-3, 74 DAFB; S3, 90 DAFB; S4, 135 DAFB. Error bars are the standard error of the mean of three replicate reactions.

The potential proanthocyanidin regulating MYB, *MYBPA1*, was expressed in both peel and flesh with transcript level correlating with the profiles of flavan-3-ols (Figure 7). In ripe fruit, where *LDOX* and *UFGT* were most up-regulated and *LAR* and *ANR* down-regulated, the transcription of *MYBPA1* was barely detectable. The other potential regulator of proanthocyanidins, *PpMYBPA2,* showed no expression in fruit (data not shown). *MYB15* and *MYB123*, both good candidates for flavonol regulation, show both skin-specificity and a decline at maturity. This correlates with the flavonol levels which are restricted to skin, early in development (Additional file 1: Table S1).

**Figure 8:**
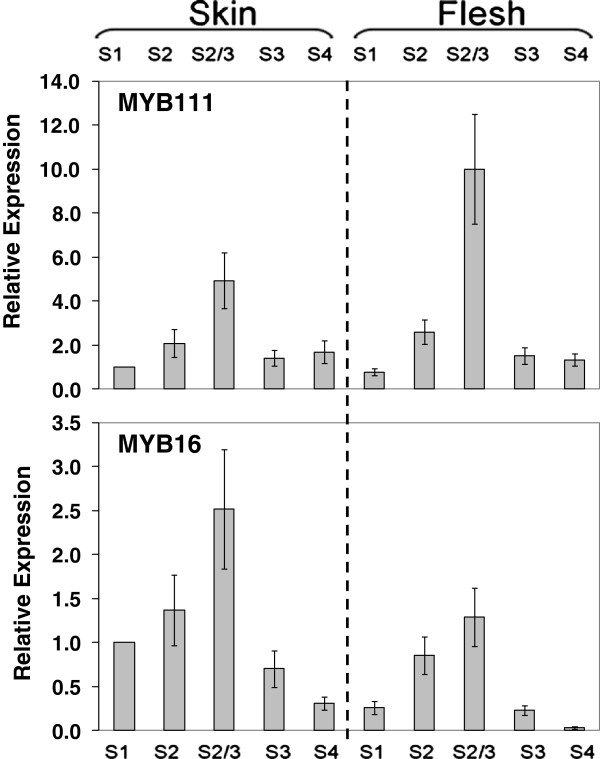
**Relative expression analyses of the transcripts of the potential MYB flavonoid repressors, *MYB111 *and *MYB16 *in peel and flesh of ‘Stark Red Gold’ nectarines during development and ripening.** The developmental stages are indicated as follows: S1, 50 DAFB; S2, 66 DAFB; S2-3, 74 DAFB; S3, 90 DAFB; S4, 135 DAFB. Error bars are the standard error of the mean of three replicate reactions.

The potential repressor MYBs, *MYB111* and *MYB16*, were expressed in both peel and flesh with transcript level correlating well with the profiles of most of the biosynthetic genes up to 90 DAFB (Figure 8). However, in ripe fruit, where *LDOX* and *UFGT* were most up-regulated, and anthocyanins are accumulated, the transcription of *MYB16* was barely detectable.

### Differential activation of nectarine biosynthetic gene promoters by MYB10 and MYBPA1

**Figure 9:**
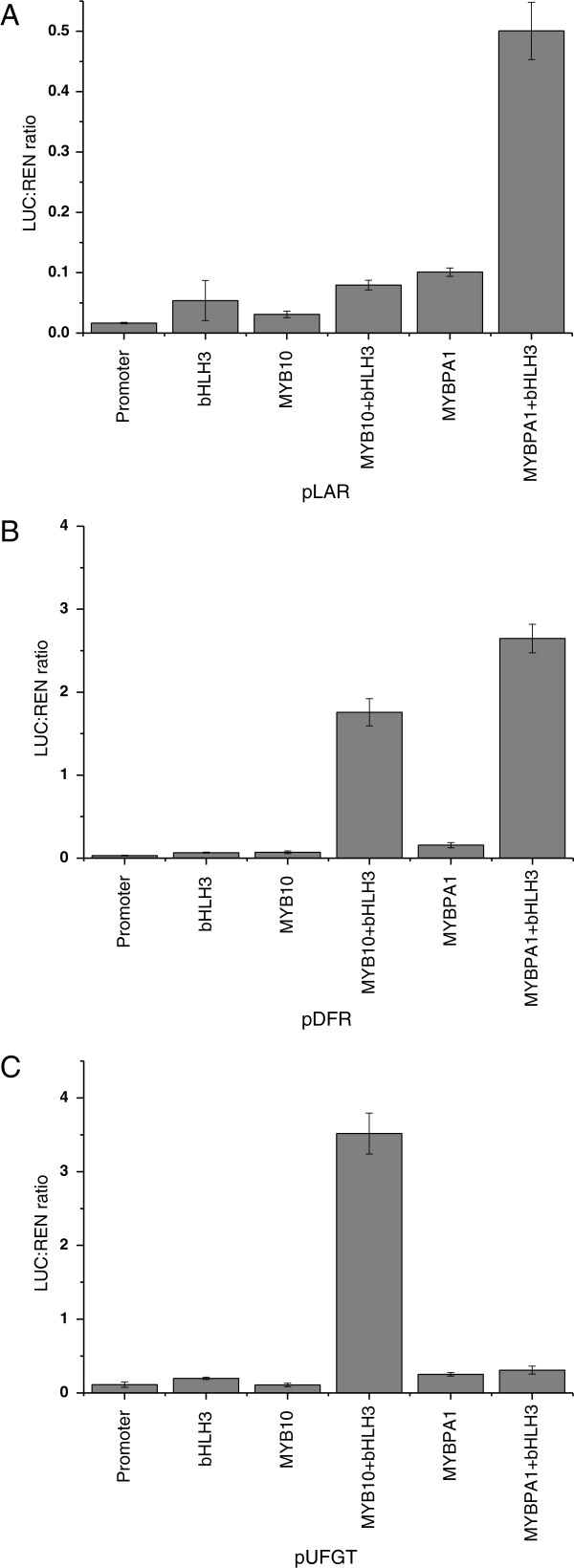
**Interaction of the peach promoters (A) *LAR*, (B) *DFR *and (C) *UFGT *by peach MYB10 and peach MYBPA1 with and without peach bHLH3 in a dual luciferase transient tobacco assay.** Leaves of *N. benthamiana* were infiltrated with the peach promoter-LUC fusion on its own (control) or co-infiltrated with MYB10 +/− bHLH3. Luminescence was measured 3 d later and expressed as a ratio of LUC to REN. Data are presented as means (± SE) of six replicate reactions.

To test the role of MYB10 and MYBPA1 on the activation of nectarine/peach flavonoid synthesis, the promoters of peach *UFGT, DFR* and *LAR* were isolated and cloned into the dual luciferase assay system [52]. *N. benthamiana* was transiently transformed by infiltration with an *Agrobacterium* suspension containing a luciferase reporter construct with fragments of each promoter and a separate *Agrobacterium* suspension containing *35S:PpMYB10, 35S:PpMYBPA1* and *35S:PpbHLH3*. While the control infiltration (promoter-LUC fusion alone) showed only minimal activity, co-infiltration of *MYB10* with *35S:PpbHLH3* showed a large rise in *UFGT* and *DFR* promoter activity with a ratio of LUC to REN of 2.5 or 2.0 respectively (Figure 9). Infiltration of the reporter with *bHLH3* or *MYB10* alone resulted in low activation. In contrast, MYBPA1 had little activity against the *UFGT* promoter, and good activity in promoting LUC expression if driven by the *DFR* and *LAR* promoters. This activation required bHLH3. *MYB10* showed little ability to activate the *LAR* promoter.

### Light-induced accumulation of anthocyanin in nectarine fruit correlates with the expression of the MYB-bHLH TFs

**Figure 10:**
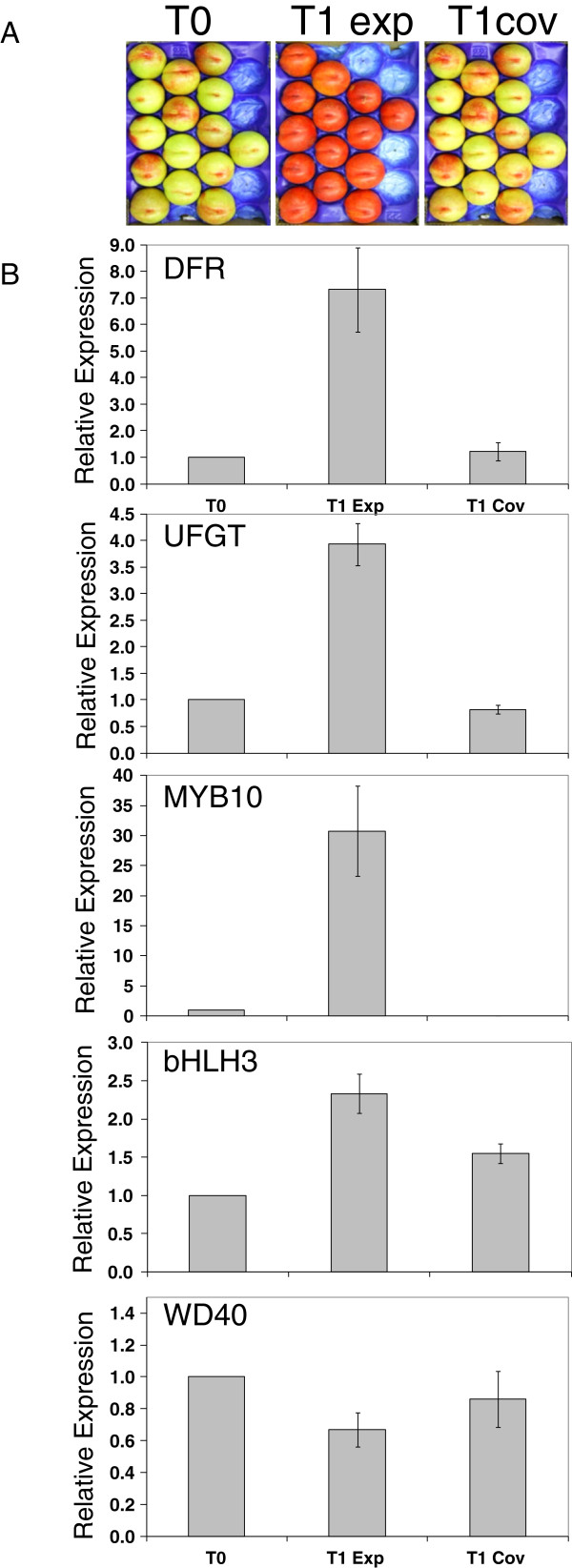
**‘Stark Red Gold’ nectarines harvested from the most shaded parts of the canopy at harvest time (T0) and after 72 hours kept under UV + white light (T1 exp) or in the dark (T1 cov) (A)., and (B) Expression analyses of the transcripts of *DFR*, *UFGT*, *MYB10*, *bHLH3 *and *WD40 *in the peel of the fruit shown in (A).** Error bars are the standard error of the mean of three replicate reactions.

Mature fruit were harvested from shaded areas of the tree, which showed low levels of colour. These fruit were either given a light treatment (UV + white light) or covered for 72 hours. Treatment with artificial light was able to strongly increase the red colouration of the peel (Figure 10A), inducing the biosynthesis and accumulation of anthocyanins. The other polyphenolic compounds, such as flavonols and proanthocyanidins showed no significant increase with UV + white light (data not shown). The influence of the light treatment on the levels of expression of *DFR*, *UFGT*, *MYB10*, *PpbHLH3* and *WD40* genes was investigated by qPCR analyses (Figure 10B). The transcripts of all the assayed genes, except the WD40, were elevated in the bright red peel of the fruit exposed to the light treatment, in comparison to low levels of transcript in the yellow peel of the fruit at harvest time and after 72 hours in the dark.

The transcript of the *DFR* and *UFGT* genes were respectively eightfold and fourfold more abundant in the light-exposed fruit than in those just harvested or kept in the dark, correlating well with the fruit peel colour. Transcripts of *MYB10*, and *bHLH3* genes were induced by the light treatment. In particular, the expression of the *MYB10* gene was the most strongly responsive to the light, showing a 30-fold elevation in the light-exposed fruit compared to the fruit at harvest time. Transcript level of *MYB10* declined to not detectable levels in the fruit kept in the dark.

Other flavonoid related genes were also analysed (Additional file 2: Figure S1). Only *FLS* showed a slight stimulation by UV/light exposure, while the regulatory MYBs *MYBPA1, MYB111* and *MYB15* were also up-regulated by the treatment.

## Discussion

### Regulation of anthocyanin and flavan-3-ol biosynthesis in nectarine/peach

The regulation of anthocyanin and flavan-3-ol biosynthesis has been studied in a diverse range of crops such as apple [27-30,39,40], grape [32,53-56], strawberry [33,42], tomato [57,58], and pepper [59]. This is due to their influence on fruit and vegetable colour, flavour and health attributes. However, there is apparently no data about the transcriptional regulation of the flavonoid metabolism in peach and nectarine (*Prunus persica*).

Our results showed that anthocyanins and flavan-3-ols have different patterns of accumulation in ‘Stark Red Gold’ nectarines during development, as observed previously [12]. Flavan-3-ols mainly accumulated at mid-season in both skin and flesh, whereas anthocyanin pigments were detected only in peel and showed peaks of biosynthesis at the beginning and end of fruit development (Figure 2B).

A similar profile for the anthocyanin accumulation was observed in the peel of ‘Flavortop’ nectarines by Tsuda et al., 2004 [48]. In this cultivar anthocyanin accumulation correlated with the transcript levels of four anthocyanin biosynthetic genes (*CHI*, *F3H*, *ANS*, *UFGT*), assayed by RNA gel-blot analysis. Our qPCR results showed that only the transcript level of the *PpUFGT* gene showed weak correlation with the accumulation of anthocyanin in fruit peel, whereas all the other flavonoid biosynthetic genes correlated with the concentration of the flavan-3-ols (Figures 3 and 4). *FLS* expression was correlated well with flavonol profile. These findings suggest that *UFGT* and *FLS* in peach fruit might be regulated more specifically than the other flavonoid genes, as observed in grape [31,32].

### Transcription factors controlling flavonoid biosynthesis

Several studies have shown that the expression of the *UFGT* gene in grape is regulated by the R2R3-MYB TF VvMYBA1, which closely correlates with anthocyanin accumulation in grape skin [54,60]. Other biosynthetic genes of the grape flavonoid pathway appear to be regulated by other R2R3-MYB TFs (VvMYBPA1, VvMYBPA2, VvMYB5a and VvMYB5b) which were recently isolated and characterized in grape [53,55]. The pattern of expression of the candidate TF genes assayed in developing ‘Stark Red Gold’ nectarines suggests a tight regulation of *UFGT* expression, which controls accumulation of anthocyanin in the peel. *LAR* and *ANR* may well be regulated by MYBPA1 in peach, while MYB15 and MYB123 are both good candidates for controlling *FLS* expression (Figure 7). Furthermore, transient expression of these candidate TFs showed that they could directly and differentially regulate promoters of the biosynthetic genes for peach polphenolics (Figure 9). The expression profiles of *MYB10*, *bHLH3* and *MYBPA1* are consistent with these TFs regulating anthocyanin and proanthocyanidins in a complex composed of both activators and repressors, as observed in *Arabidopsis*[43-45]*.*

This regulatory complex, composed of MYB10, bHLH3 and WD40, might control the biosynthesis of the anthocyanin in peach, and the competing presence of MYBPA1 may explain the induction of flavan-3-ols. Evidence for this comes from peach flesh, where no *MYB10* expression was detected (Figure 6), yet abundant expression of *MYBPA1* and all the genes except *UFGT* is seen (Figures 3 and 7). There is accumulation of flavan-3-ols suggesting a functioning pathway until at least *LDOX*. Peach MYBPA1 was shown to be able to activate the *DFR* and *LAR* promoters (Figure 9).

### Light-induced anthocyanin accumulation in peach skin is regulated by MYB-bHLH complex

A previous study conducted by Kataoka and Beppu., 2004 [61] showed that anthocyanin accumulation in peach peel was enhanced by UV light. Our expression analyses of the *UFGT*, *MYB10*, and *bHLH3* genes in the peel of ‘Stark Red Gold’ nectarines under different light conditions suggest that the regulatory complex described above might be responsive to environmental stimuli (Figure 10).

Flavonol accumulation was not significantly affected by light, although the 72 hour treatment may be too short for this compound class. The accumulation of anthocyanin in the peel of the light-exposed fruit correlated with enhanced transcript levels of all the assayed genes. In particular, the *MYB10* gene was the most strongly responsive to the light, with transcripts not detectable in the fruit kept in the dark and 30-fold more abundant in the light-exposed fruit compared to the fruit at harvest time. These results suggest that anthocyanin biosynthesis was not activated in the fruit kept in the dark because of the absence of MYB10. In the light-exposed fruit, instead, abundant MYB10 might be able to form the protein complex for the induction of the *UFGT* expression even in the presence of repressors such as MYB111 and MYB16 (Figure 8), as suggested by Matsui et al., (2008) [45] in studies using *Arabidopsis*. In *Arabidopsis* the repressor AtMYBL2 is involved in a positive regulatory loop with the bHLH TT8 and R2R3 MYB PAP1 [44,45], so up-regulation of peach *MYB111* by the activation complex may also be occurring.

## Conclusions

The patterns of accumulation of flavonols, flavan-3-ols and anthocyanins in developing ‘Stark Red Gold’ nectarines suggest that the biosynthesis of these phenolic compounds is differentially regulated in peach/nectarine fruit. The expression levels of the main flavonoid biosynthetic genes confirmed this hypothesis. The *UFGT* gene transcript level correlates with anthocyanin accumulation in peach fruit, whereas the patterns of expression of all the other biosynthetic genes correlate with the concentration of flavan-3-ols. These patterns of gene expression might be explained by the involvement of different transcription factors. In particular, we showed that a regulatory complex composed of a MYB, bHLH3 and WD40 controls promoters of polyphenolic biosynthetic steps in peach fruit, activating *UFGT* (MYB10 and bHLH3) or *LAR* (MYBPA1 and bHLH3) gene transcription.

The characterization of these transcription factors involved in anthocyanin regulation provides essential information for peach fruit breeding programs with the final aim of selecting new cultivars with improved aesthetical and nutritional properties.

## Methods

### Plant material

Yellow-fleshed nectarines cv. ‘Stark Red Gold’ were collected during the growing season (from May 2008 until August 2008) from an orchard located at the Cadriano Experimental Station (University of Bologna, Italy; 44° 32´ 54.098" N - 11° 23´ 13.226" E). Peel (including about 1 mm of the cortical tissue) and flesh samples were collected separately from at least 50 replicate fruit at each of the following developmental stages: S1, 50 days after full bloom (DAFB); S2, 66 DAFB; S2/S3, 74 DAFB; S3, 90 DAFB; S4, 135 DAFB.

For analysis of the effect of light on pigmentation change 45 ripe ‘Stark Red Gold’ nectarines were harvested at 138 DAFB from the most shaded areas of the tree canopy, to select fruit with a low concentration of anthocyanin. Skin samples were collected from 15 fruit at harvest time (T0). The remaining 30 fruit were transferred to a growth chamber at a constant 25°C, with UV and white light lamps providing 56 μW/cm^2^ of UVA, 30 μW/cm^2^ of UVB and 7000 lux at fruit surface. Fifteen fruit were completely covered by aluminium foil, whereas the other 15 were exposed to the light. Skin samples were collected after 72 hours from both the fruit exposed to the light (T1 exposed) and those kept in the dark (T1 covered).

At sampling time all the fruit tissues were immediately frozen in liquid nitrogen and stored at −80°C until RNA or phenolic compounds were extracted.

### Identification and quantification of anthocyanins, flavonols and flavan-3-ols

Fruit samples (separated as peel and flesh) were freeze-dried and ground with pestle and mortar to a fine homogeneous powder. Powder (100 mg dry weight) was then extracted with 1 mL methanol containing 6-methoxy-flavone (0.025 mg mL^-1^ in methanol) as an internal standard. Extractions were performed for 30 min in an ultrasonic bath, the water being cooled with ice, followed by centrifugation at 12500 g for 20 min at 0°C. The supernatant was then collected and analysed by high-performance liquid chromatography (HPLC).

Samples were analysed by a Waters HPLC system with a Photodiode Array Detector (Waters 2996) and a reverse-phase Supelcosil™ LC-18 HPLC column (15 cm long, 4 mm internal diameter and octadecyl silane particles of 5 μm diameter), as described previously [12].

Anthocyanins, flavonols, and flavan-3-ols were identified by comparison of the retention times and UV spectra (between 210 and 560 nm wavelength) with authentic standards of cyanidin 3-*O*-glucoside, cyanidin 3-rutinoside, quercetin 3-galactoside, quercetin 3-glucoside, quercetin 3-rutinoside, catechin and epicatechin. Co-chromatography was applied to further confirm initial preliminary identification of chromatographic peaks. Concentrations of anthocyanins and flavan-3-ols, expressed in mg g^-1^ dry weight (DW), were calculated from calibration curves obtained with the corresponding external standards. All standards were acquired from Sigma-Aldrich (St Louis, MO, USA).

### Real-Time qPCR expression analysis

RNA was isolated from the fruit (peel and flesh separately) by a method adapted from that described by Chang et al., (1993) [62]. Following DNaseI treatment using an Ambion DNA-free™ kit, first-strand cDNA synthesis was carried out on 2 ug of RNA using anchored-oligo(dT)_18_ and random hexamer primers according to the manufacturer’s instructions (Transcriptor First Strand cDNA synthesis kit; Roche Diagnostic, Mannheim, Germany).

Genes encoding peach flavonoid pathway enzymes and regulators were selected by homology from the GenBank database and the Genome Database for Peach (http://www.rosaceae.org/species/prunus_persica/genome_v1.0; draft kindly made available by the International Peach Genome Initiative). Gene-specific primers (Table 1) corresponding to these genes were designed using Vector NTI version 9.0.0 (http://www.invitrogen.com) to a stringent set of criteria, enabling application of universal reaction conditions. To check reaction specificity, RT-PCR reactions were carried out according to the manufacturer’s instructions (Platinum Taq, Invitrogen, Carlsbad, CA, USA), with a thermal profile as follows: pre-incubation at 95°C for 5 min followed by 35 cycles of 95°C (10 sec), 56°C (20 sec) and 72°C (30 sec), with a final extension at 72°C for 7 min. The sequences of each primer pair and the relevant accession numbers are shown in Table 1.

**Table 1:** Forward and reverse primers for peach genes used in qPCR analysis of biosynthetic enzymes and candidate transcription factors

**Gene**	**Accession No./Prunus gene model**	**Real time PCR primers (5’ to 3’)**	
		**Forward**	**Reverse**
**Biosynthetic genes:**			
***CHS***	**AB094986**	CAGAGATACCCAAAGGTTGGAAGGC	AACCATCCTTCCCGACAGCGAT
	**ppa006888m**		
***CHI***	**DY634915**	TGAAGACCTCAAGGAACTTCTCAATGG	ACACAGGTGACAACGATACTGCCACT
	**ppa011276m**		
***F3H***	**AB097151**	TCCGAGGGCAGAGCGAAGAAC	TTGTGGAGGCTTGTGAGGATTGG
	**ppa007636m**		
***DFR***	**AB095030**	GGTCGTCCAGGTGAACATACTGCC	ATTTCTCATGCCATCCATGCCAC
	**ppa008069m**		
***LDOX***	**EU292219**	AAGTGGGTCACTGCCAAGTGTGTTC	GTGGCTCACAGAAAACTGCCCAT
	**ppa007738m**		
***UFGT***	**DN676790**	CCGCTGCCTCTCCCAACACTC	CCATCAGCCACATCAAACACCTTTAT
	**ppa005162m**		
***ANR***	**AM288300**	ACTTCAAGGCTAAGGGGCTGCTG	CCAAGCCAGATAAACGCCAATCAC
	**ppa008295m**		
***LAR***	**AJ872926**	CATCCACGGGGAAATTCACCTG	ACCCTTCCCAGAGTTACCATCACTGA
	**ppa007994m**		
***FLS1***	**GU938685**	GTTTTCTGACGGCAACGTTACGAA	CCCAACCCTAGCGATAGGAGCC
	**ppa008322m**		
**Regulators:**			
***MYB10***	**EU155160**	TGATTCCAAGGGTCCACGCTAAAA	CTGGTCTTGGGTTAGATGAAGAACTGC
	**ppa016711m**		
***bHLH3***	**DY642427**	TTCCTCTACTAGACGGCGTCGTCG	GGAGGAGGATGGTGGTTGTGGTC
	**ppa002884m**		
***WD40***	**ACQ65867**	CCCAGCCTGATACCCCTTTGCT	GTCGGCGAACGGATATCCAAAAT
	**ppa008187m**		
***MYBPA1***	**CV047374**	GACCCAAGCACCCACAAGAAATTATC	GGCTTTAGTGGCTCCACATGTTGA
	**ppa009439m**		
***MYB15***	**ppa004560m**	GGAAAATTGACACCTTCAGAAGGCC	TCTACCGCCTCTTCGCTTGGAA
***MYB16***	**ppa010277m**	AGCTTTTGACCAGAGGTATCGACCC	TGCCCAGAAGCCCATTAATAATGC
***MYB111***	**ppa010716m**	CGCTTATTGCTGGAAGGTTGCC	GCCTATGGTTATTGGGATCAATGCC
***MYB123***	**ppa023768m**	ACTCAACCGTCGACCAACATTGC	TCGGCGTGGTGAGGAAAGATG
**Reference gene:**			
***PpActin***	**BU045718**	GGAAATTACTGCATTAGCACCCAGC	CCAGATTCATCATACTCGGCTTTGG
	**ppa007214m**		

qPCR DNA amplification and analysis was carried out using the LightCycler System (Roche LightCycler® 480; Roche Diagnostics). All reactions were performed using the LightCycler® 480 SYBR Green I Master Mix (Roche Diagnostics) according to the procedure described by the manufacturer. Reactions were performed in triplicate using 2.5 μl 2 × Master Mix, 0.25 μl each primer (10 μM), 1.25 μl diluted cDNA (1:50) and nuclease-free water (Roche Diagnostics) to a final volume of 5 μl. A negative water control was included in each run. Fluorescence was measured at the end of each annealing step. Amplification was followed by a melting curve analysis with continual fluorescence data acquisition during the 65–95°C melt. The raw data were analysed with the LightCycler software, version 4, and expression was normalized to *Prunus persica* actin (Actin; BU045718) to minimize variation in cDNA template levels. Actin was selected for normalization due to its consistent transcript level throughout fruit tissues, with crossing threshold (Ct) values changing by < 2. The skin sample at 50 DAFB (S1) for the developmental series and that at harvest time (T0) for the experiment with artificial light were selected as calibrators with nominal value of 1. For each gene, a standard curve was generated using a cDNA serial dilution, and the resultant PCR efficiency calculations (ranging between 1.849 and 1.989) were imported into relative expression data analysis. Error bars shown in qPCR data are technical replicates, representing the means ± SE of three replicate qPCR reactions.

### Statistical analysis

Gene expression levels were correlated with anthocyanin, proanthocyanidin and flavonol concentrations over a fruit development series. Pearson correlation (r) analysis was performed and tested for statistical significance.

All statistical analysis was carried out using Minitab® 16.1.1 statistical software (Minitab, Inc. (2009). Minitab Statistical Software, Release 16 for Windows, State College, Pennsylvania. Minitab® is a registered trademark of Mintab, Inc).

### Transient expression of candidate transcription factors

Upstream regions from the ATG start site of the peach genes *UFGT* (2 kb)*, DFR* (1.6 kb)*,* and *LAR* (1.4 kb) were isolated from peach gDNA by PCR. These promoter fragments were inserted into the cloning site of pGreen 0800-LUC [28,52]. In the same construct, a luciferase gene from *Renilla* (REN), under the control of a 35S promoter, provided an estimate of the extent of transient expression. Activity was expressed as a ratio of LUC to REN activity. The promoter-LUC fusions were used in transient transformation by mixing 100 μl of *Agrobacterium* strain GV3101 (MP90) transformed with the reporter cassette with or without another *Agrobacterium* culture(s) (450 μl) transformed with a cassette containing *MYB10, bHLH3* and/or *MYBPA1* fused to the 35S promoter. The *Agrobacterium* strains carrying the promoter-*LUC* fusions and transcription factors were used in transient transformation of *Nicotiana benthamiana* leaves after co-inoculation and 3 days transient transformation.

## Competing interests

The authors declare they have no competing interests.

## Authors’ contributions

DR conceived of the study, participated in its design, carried out the orchard and molecular biology experiments and drafted the manuscript, ACA and GC conceived of the study, participated in its design and coordination and helped draft the manuscript, RVE and RAH-K carried out DNA and RNA extractions and real-time experiments, CA and VZ carried out flavonoid quantification analysis and edited the manuscript, RPH participated in the design of the study and helped draft the manuscript. All authors read and approved the final manuscript.

## Supplementary Material

Additional file 1: Table S1 Table showing Pearson's correlation coefficient (a) and p-values (b) for individual gene expression profiles compared to metabolic data. Click here for file

Additional file 2: Figure S1 ‘Stark Red Gold’ nectarines harvested from the most shaded parts of the canopy at harvest time (T0) and after 72 hours kept under UV + white light (T1 exp) or in the dark (T1 cov) as Figure 10. Expression analyses of the transcripts of *FLS1, LAR, ANR, MYBPA1, MYB111* and *MYB15* in the peel of the fruit. Error bars are SE for three replicate reactions. Click here for file
