# Supplementary material for: Transcriptional regulation of flavonoid biosynthesis in nectarine (Prunus persica) by a set of R2R3 MYB transcription factors
Source: BMC Plant Biol. 2013 Apr 25;13:68. doi: 10.1186/1471-2229-13-68 (PMC3648406; doi:10.1186/1471-2229-13-68)
Supplement: Additional file 1: Table S1 — Table showing Pearson's correlation coefficient (a) and p-values (b) for individual gene expression profiles compared to metabolic data. [file 1471-2229-13-68-S1.docx]

Table S1

| Gene | Flavan-3-ols | Anthocyanins | Flavonols |
| --- | --- | --- | --- |
| PpCHS | ^a^0.475 | -0.581 | -0.373 |
|  | ^b^0.419 | 0.304 | 0.536 |
| PpCHI | 0.593 | -0.758 | -0.568 |
|  | 0.292 | 0.138 | 0.318 |
| PpF3H | 0.454 | -0.623 | -0.39 |
|  | 0.443 | 0.262 | 0.517 |
| PpDFR | 0.796 | -0.791 | -0.556 |
|  | 0.107 | 0.111 | 0.33 |
| PpANR | 0.475 | -0.669 | -0.428 |
|  | 0.419 | 0.217 | 0.473 |
| PpLAR1 | 0.473 | -0.75 | -0.494 |
|  | 0.421 | 0.144 | 0.398 |
| PpFLS | -0.638 | 0.757 | 0.895 |
|  | 0.247 | 0.139 | 0.04 |
| PpLDOX-1 | 0.621 | -0.802 | -0.896 |
|  | 0.264 | 0.102 | 0.04 |
| PpUFGT | -0.425 | 0.334 | -0.019 |
|  | 0.476 | 0.583 | 0.975 |
| PpMYB10 | -0.012 | -0.438 | -0.199 |
|  | 0.985 | 0.461 | 0.749 |
| bHLH3 | 0.308 | -0.600 | -0.754 |
|  | 0.614 | 0.285 | 0.141 |
| PpMYBPA1 | 0.618 | -0.609 | -0.303 |
|  | 0.266 | 0.276 | 0.62 |
| PpWD40 | 0.389 | -0.622 | -0.511 |
|  | 0.518 | 0.262 | 0.379 |
| PpMYB15 | -0.613 | 0.526 | 0.705 |
|  | 0.272 | 0.362 | 0.183 |
| PpMYB123 | -0.661 | 0.963 | 0.994 |
|  | 0.224 | 0.008 | 0.001 |
| PpMYB16 | 0.084 | -0.212 | 0.034 |
|  | 0.893 | 0.732 | 0.957 |
| PpMYB111 | 0.255 | -0.462 | -0.331 |
|  | 0.679 | 0.433 | 0.587 |

Pearson's correlation coefficient (a) and p-values (b) for individual gene expression profiles compared to metabolic data.
