# Supplementary figures and images for: Transcriptional regulation of flavonoid biosynthesis in nectarine (Prunus persica) by a set of R2R3 MYB transcription factors
Source: BMC Plant Biol. 2013 Apr 25;13:68. doi: 10.1186/1471-2229-13-68 (PMC3648406; doi:10.1186/1471-2229-13-68)

## Slide 1
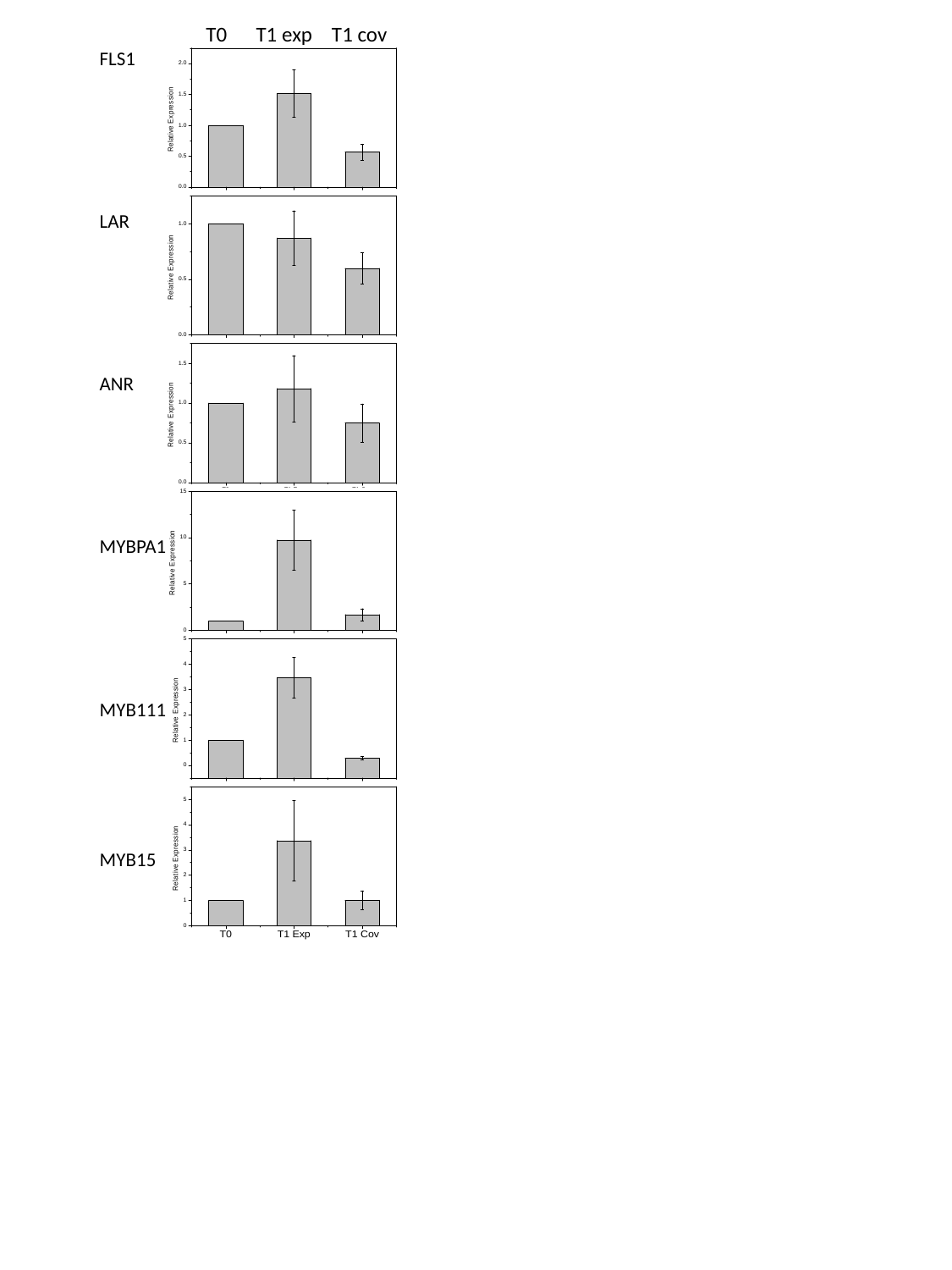

T0 T1 exp T1 cov
FLS1
LAR
ANR
MYBPA1
MYB111
MYB15

Supplement: Additional file 2: Figure S1 — ‘Stark Red Gold’ nectarines harvested from the most shaded parts of the canopy at harvest time (T0) and after 72 hours kept under UV + white light (T1 exp) or in the dark (T1 cov) as Figure 10. Expression analyses of the transcripts of FLS1, LAR, ANR, MYBPA1, MYB111 and MYB15 in the peel of the fruit. Error bars are SE for three replicate reactions. [file 1471-2229-13-68-S2.pptx]
